# Development of Two Murine Antibodies against *Neospora caninum* Using Phage Display Technology and Application on the Detection of *N. caninum*

**DOI:** 10.1371/journal.pone.0053264

**Published:** 2013-01-08

**Authors:** Jinhua Dong, Takahiro Otsuki, Tatsuya Kato, Tetsuya Kohsaka, Kazunori Ike, Enoch Y. Park

**Affiliations:** 1 Laboratory of Biotechnology, Graduate School of Science and Technology, Shizuoka University, Shizuoka, Japan; 2 Laboratory of Biotechnology, Department of Applied Biological Chemistry, Faculty of Agriculture, Shizuoka University, Shizuoka, Japan; 3 Laboratory of Animal Reproduction & Physiology, Faculty of Agriculture, Shizuoka University, Shizuoka, Japan; 4 Laboratory of Veterinary Parasitology, Nippon Veterinary and Life University, Musashino, Tokyo, Japan; Federal University of Pelotas, Brazil

## Abstract

Neosporosis, caused by an intracellular parasite, *Neospora caninum*, is an infectious disease primarily of cattle and dogs. It occurs worldwide and causes huge damages to dairy farms. In this study, we immunized mice with recombinant surface-associated protein 1 of *N. caninum* (rNcSAG1) and developed two novel monoclonal antibodies, A10 and H3, against NcSAG1 using phage-display technology. Both clones bound to purified rNcSAG1 and the half maximal inhibitory concentrations of A10 and H3 are 50 and 72 nM of rNcSAG1, respectively. In immunofluorescence assays, both A10 and H3 Fabs bound to *N. caninum* parasites. Direct detection of *N. caninum* parasites was developed firstly using an enzyme-linked immunosorbent assay (ELISA) with A10 and H3. Binding of A10 and H3 antibodies to rNcSAG1 was also inhibited by some certain anti-*N. caninum* antibodies in the neosporosis-positive cattle sera, suggesting they might bind to the same epitopes of NcSAG1 with those anti-*N. caninum* antibodies of bovine. These antibodies were demonstrated to have a potential for monitoring the *N. caninum* parasites in a dairy farm, which may lead to protect livestock from parasite-infection.

## Introduction

Neosporosis is an infectious disease primarily of cattle, caused by *Neospora caninum*. Neosporosis now appears to be a major cause of abortion in dairy cattle worldwide [1]. *N. caninum* is an obligate intracellular protozoan parasite, which was first recognized in dogs in Norway [2] and has been found to infect a wide variety of mammals such as cattle, sheep, goats, deer, and horses [3]–[5].

For diagnosis of neosporosis, various methods have been developed. The indirect fluorescent antibody test (IFAT) was employed to detect anti-*N. caninum* antibodies in sera of cattle and to evaluate the infection status [6]–[8]. Besides IFAT, other serological diagnostic tools, such as immunoblotting [9], agglutination tests [10], and enzyme-linked immunosorbent assays (ELISAs) [11]–[13], are also available. However, most of these methods focus on detection of anti-*Neospora* antibodies in cattle serum, and none was designed to detect parasite in the meal of cattle or field. Furthermore, there is no effective method of control or medical treatment of neosporosis. Monitoring the *N. caninum* parasites to reduce the likelihood of infection in a farm is an urgent issue for protection.

Proteins displayed on the surfaces of intracellular pathogens are believed to play critical roles in infection. The surface-associated protein 1 of *N. caninum* (NcSAG1) has been identified as one of major surface antigens of *N. caninum* tachyzoites and demonstrated to be immune dominant and involved in interactions between the tachyzoite and the host cell [14]. Its predominant antigenicity was also demonstrated by its recognition by antisera from *Neospora-*infected animals [15].

Antibodies, produced by B cells in vertebrata, are used by the immune system to identify and neutralize foreign objects such as bacteria and virus. Because antibody recognizes a unique part of the foreign target [16], it has been used in many fields such as detection, imaging, and drug developments. Hybridoma technology is a method of forming hybrid cell lines by fusing a specific antibody-producing B cells with a myeloma cell for production of monoclonal antibodies [17]. However, some other technologies may be needed to analyze antibody gene in hybridoma. Phage display technology is a powerful tool to generate antibodies recognizing specific antigens [18]. It includes a number of crucial steps, such as the creation of diversity, coupling of phenotype to genotype, selection, amplification, and analysis.

In this paper, we report the immunization of mice with recombinant *N. caninum* proteins NcSAG1(rNcSAG1) from silkworm larvae and development of the *N. caninum-*specific antibodies from mice by employing phage display technology. Direct detection of *N. caninum* parasites was developed using those *N. caninum-*specific antibodies.

## Results

### Immunization of Mice with rNcSAG1

Mice were immunized with purified rNcSAG1 that was expressed from silkworm larvae, and total RNA was extracted from spleen cells of immunized mice. After transcription of cDNA, the variable region genes of heavy chain (V_H_) and light chain (V_L_) of antibodies were amplified using polymerase chain reaction (PCR). PCR products were cloned into a phagemid and transformed *E. coli* to make antibody displaying phage library from which anti-NcSAG1 antibodies are screened (Figure S1).

Inbred BALB/c mice were immunized with rNcSAG1. After one week of final immunization, blood of immunized mice was taken by tale bleeding and anti-NcSAG1 antibodies in sera samples were confirmed with an indirect enzyme-linked immunosorbent assay (ELISA). High-signal intensity was observed in the wells on which rNcSAG1 was immobilized, and only a low signal was detected for bovine serum albumin (BSA) which was also immobilized on microplate as a negative control. The signals against rNcSAG1 decreased when the sera were diluted (Figure S2). This demonstrates that the mice were immunized with rNcSAG1 successfully and antibodies against the rNcSAG1 contained in the sera of mice.

### Monoclonal Antibody Selection from Phage Display Library

**Figure 1:**
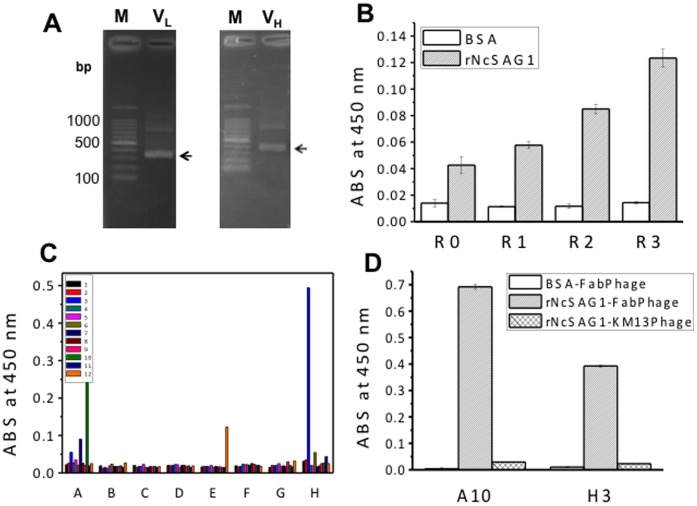
Selection of monoclonal antibodies. (A) Amplified genes of antibody variable regions. The arrows indicate the target DNA bands. (B) Enrichment of rNcSAG1-specific Fab-phage. rNcSAG1 (0.5 µg/ml), and BSA (10 µg/ml) were immobilized on a 96-well microplate, respectively. ECL™ Anti-mouse IgG, Horseradish Peroxidase linked whole antibody (from sheep) was used as the secondary antibody. R0 stands for the original phage library, whereas R1, R2, and R3 stand for the amplified Fab-phage in rounds 1, 2, and 3 of biopanning, respectively. Experimental data were presented as average values with standard error (n = 3). (C) Screening of monoclonal Fab-phage. (D) Binding of Fab-phages to BSA and rNcSAG1.

The advantage of the phage display system is the coupling of a selectable function (binding to an antigen) to the genetic material that encodes that function. The display of the Fab fragment was developed using pDong1/Fab with the help of the KM13 helper phage, which allows specific recovery of antigen-binding phages by protease treatment [19]. For construction of antibody library, the V_H_ and V_L_ genes of antibodies were amplified and their DNA fragments detected at 350–400 bp were considered target genes (Figure 1A). A phage display antibody library with a diversity of 5×10^5^ was obtained using the phagemid pDong1/Fab. After three rounds of selection, the enrichment of NcSAG1-binding phage was confirmed using an ELISA with original phage library R0 and sublibraries R1, R2, and R3 that were amplified in each step of biopanning. Absorbance at 450 nm in phage ELISA for R0, R1, R2, and R3 phage against rNcSAG1 increased with the increase of biopanning step (Figure 1B), suggesting that three rounds of biopanning enriched the rNcSAG1-specific Fab-phages. Those R0–R3 phage did not bind to BSA as a negative control.

Phages obtained in the third round were used to infect *E. coli* TG-1 for forming colonies. Ninety-six colonies were picked up, cultivated for forming phage. Two clones A10 and H3 showed strong signal against immobilized rNcSAG1 (Figure 1C). The binding capacity of A10 and H3 Fab-phage was also reconfirmed against rNcSAG1. A10 and H3 clones bound to rNcSAG1 but not to BSA (Figure 1D), suggesting their specificity to rNcSAG1. The KM13 helper phage on which no antibody was displayed was used as a control and did not bind to immobilized NcSAG1.

### IC_50_ of A10 and H3 Clones

**Figure 2:**
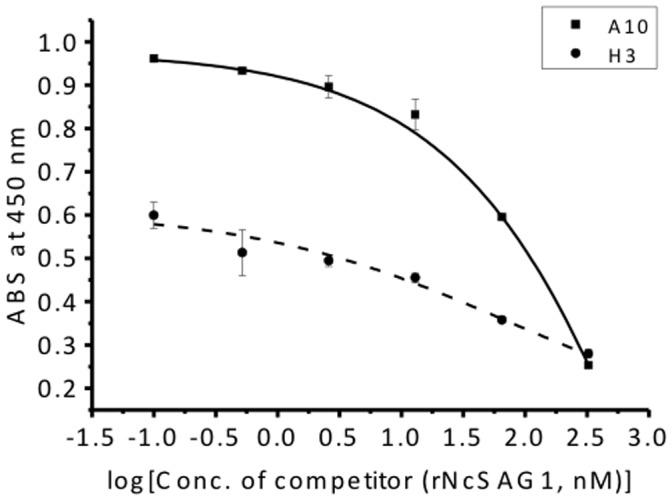
The half maximal inhibitory concentration of A10 and H3 to rNcSAG1. rNcSAG1 solutions with serially diluted concentration were used to inhibit the binding of Fab-phage of A10 and H3 to immobilized rNcSAG1 on a microplate. The IC_50_ of both clones was evaluated to be 50 and 72 nM of rNcSAG1 based on the dose-response curve (n = 3).

The half maximal inhibitory concentration (IC_50_) is a measure of the effectiveness of a compound in inhibiting biological or biochemical function. To evaluate the IC_50_ of those two clones, a competitive ELISA with serially diluted rNcSAG1 solutions that inhibit the binding of Fab-phage to immobilized rNcSAG1 was performed. Competition was observed between free and immobilized rNcSAG1, and the IC_50_ of A10 and H3 was evaluated to be 50 and 72 nM of rNcSAG1, respectively (Figure 2).

### Purification of Fab Antibodies and their Binding to rNcSAG1

**Figure 3:**
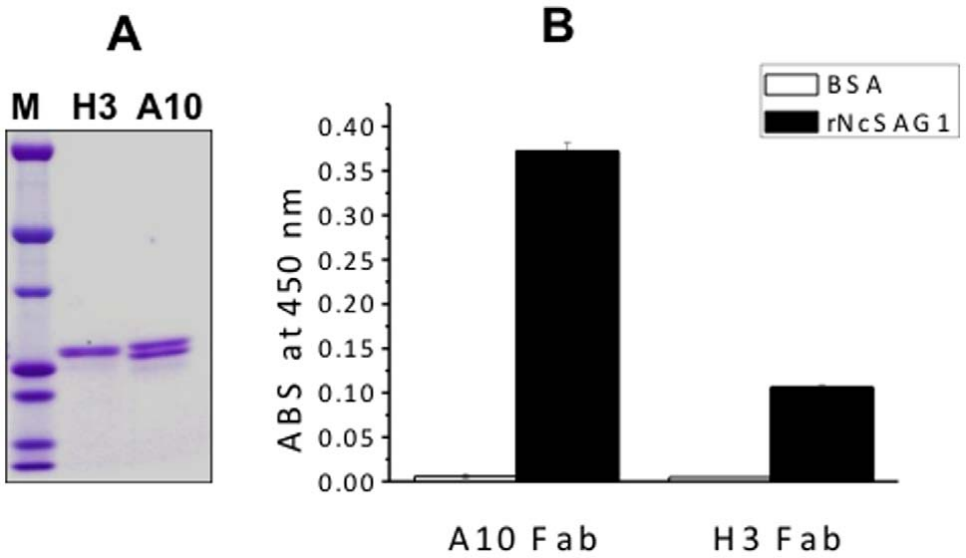
Purification of Fab antibodies and binding assays to rNcSAG1. SDS-polyacrylamide gel electrophoresis analysis of purified Fab antibodies (A) and the binding of A10 and H3 to rNcSAG1 proteins (B). M: Precision Plus Protein™ Dual Colors Standards. Anti-Nc ab: anti-*Neospora caninum* antibody.

Fab antibodies were expressed in *E. coli* and purified with TALON Co^2+^-immobilized resin and an Anti-FLAG M2 affinity gel. The purified Fab fragments showed two bands on sodium dodecyl sulfate polyacrylamide gel electrophoresis (SDS-PAGE) with molecular weight of 24 and 26 kDa, respectively (Figure 3A), which were identified as light chain and V_H_-C_H_1 of heavy chain. The binding of these Fabs to rNcSAG1 was confirmed with an ELISA (Figure 3B). Both Fabs did not bind to BSA as a control.

### Immunofluorescence Assay of *N. caninum* with Monoclonal Antibodies

**Figure 4:**
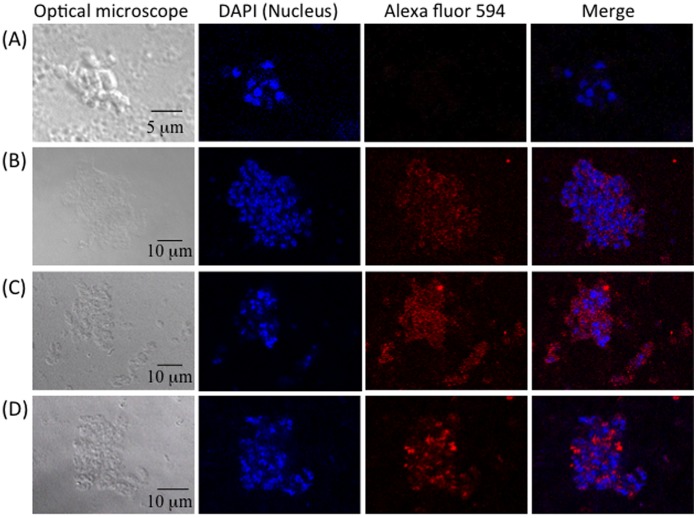
Immunofluorescence assay of *N. caninum* with monoclonal antibodies. *N. caninum* was immobilized and blocked following by addition of sample without primary antibody (A), A10 Fab (B), H3 Fab (C), and a commercial anti-*Neospora* antibodies (D). For Fab samples, anti-FLAG antibody was used as a secondary antibody. Alexa 594 labeled anti-mouse IgG was added finally and observed under a confocal laser-scanning microscope. 4',6-diamidino-2-phenylindole (DAPI) was used to stain the nuclear of parasites.

To confirm whether A10 and H3 Fabs bind to *N. caninum*, an immunofluorescence assay was performed using a commercial anti-*Neospora* antibody as a positive control. Under confocal laser microscopy, parasites’ nuclear were identified with DAPI-staining, showing blue fluorescence (Figure 4). Staining with the A10 and H3 Fabs and Alexa Fluor 594-labeled antibodies (Figure 4B and C) revealed red fluorescence, suggesting both A10 and H3 Fabs bound to parasites as the commercial anti-*Neospora* antibody did (Figure 4D). For the negative control without primary antibody, no fluorescence was observed (Figure 4A).

### Detection of *N. caninum* Parasites in a Parasite Solution

**Figure 5:**
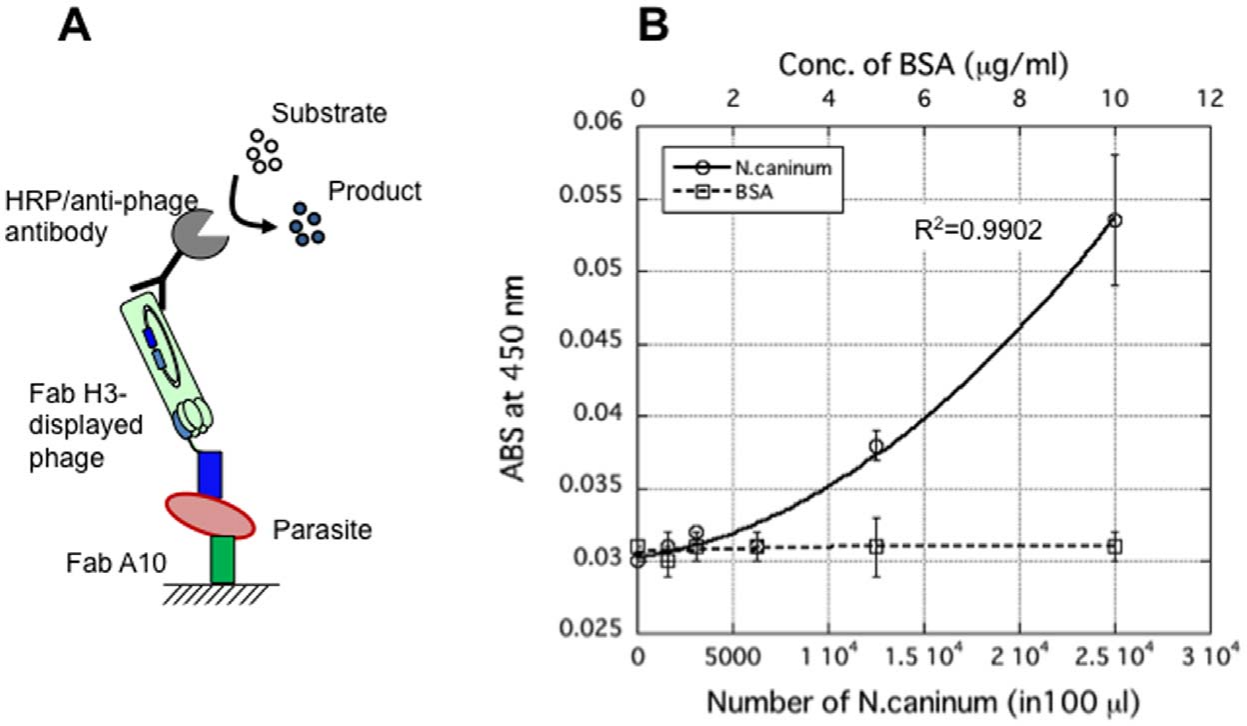
Direct detection of parasites in a sandwich ELISA. Antibody A10 was immobilized on the surface of microplate and then parasites was added. After washing, Fab-H3 displayed phage was added and A10, parasite, phage-H3 form a complex, and immobilized on the surface by A10. HRP/anti-M13 monoclonal antibody conjugate was used to detect the complex. (A) Schematic diagram for detection of parasites. (B) Correlation between parasite and Fabs with a correlation factor of 0.9902 (n = 3). Bovine serum albumin (BSA) was used as a negative control.

For a direct detection of *N. caninum* in a parasite solution, a sandwich ELISA was developed (Figure 5A). A10 Fab was immobilized on the surface of a microplate and serially diluted parasite solution was then added to each well and incubated. After washing the microplate, Fab H3-displaying phage was added and the H3-parasite-A10 complex on the surface of wells was detected by Horseradish peroxidase (HRP)/anti-M13 monoclonal antibody conjugate. The absorbance was increased with the increase of the number of parasite in solution, and a correlation between Fab and parasite number was fitted with correlation coefficient (R^2^) of 0.9902 (Figure 5B). The number of parasite in solution was counted by microscopic observation of *N. caninum* in solution, which was used for a standard curve. In the control samples with different concentrations of BSA, no significant signal increase was observed. This suggests that the developed Fabs can be used for the detection of *N. caninum* parasites in solution and the limit of detection (LOD) was estimated to be around 6.333×10^3^ parasites in 100 µl PBS solution.

### Inhibition of rNcSAG1 Binding of Murine Anti-rNcSAG1 Antibody Clones with Cattle anti-*N. caninum* Antibodies

**Figure 6:**
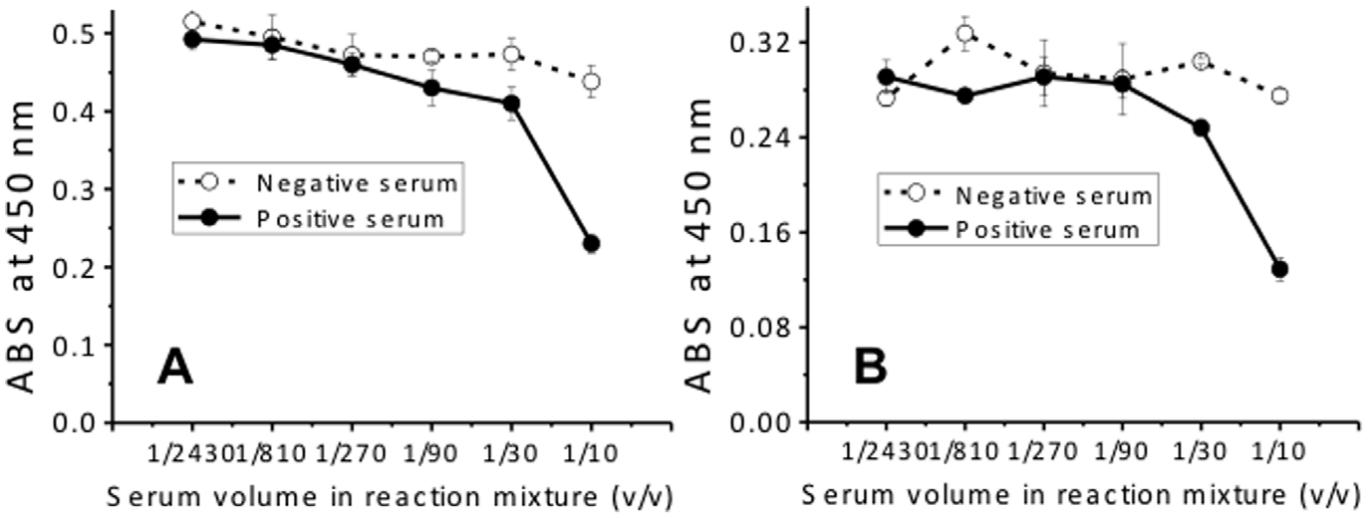
Inhibition of A10 (A) and H3 (B) antibody binding to rNcSAG1 with anti-*N. caninum* antibodies in neosporosis-positive serum. Sera samples from healthy cattle (negative) and that from cattle with neosporosis (positive) were used to inhibit the binding of A10 or H3 to immobilized rNcSAG1on a microplate (n = 3).

Anti-*N. caninum* antibodies including anti-NcSAG1 antibodies are generally produced by the adaptive immune system of cattle when they are infected with *N. caninum*. Therefore, another competitive ELISA was also performed to check the competition between Fab-phage and anti-*N. caninum* antibodies in neosporosis-positive cattle serum. The binding of A10 and H3 to rNcSAG1 was inhibited with the increase in the ratio of neosporosis-positive serum (Figure 6A and B). For a negative serum, no significant signal increase was observed. To check the reproducibility of this result, another pair of sera containing one negative and one positive samples, which were provided by Tobu Livestock Diagnostic Center of Shizuoka Prefecture of Japan and evaluated with a commercial diagnostic kit, was also tested. The Bindings of A10 and H3 to rNcSAG1 were also inhibited (Figure S3), even though the signal difference was limited. These results suggest that A10 and H3 antibodies might bind to the same epitopes with some certain bovine anti-*N. caninum* antibodies in cattle serum.

### Sequences and Structures of Murine Anti-*Neospora* Antibodies

**Figure 7:**
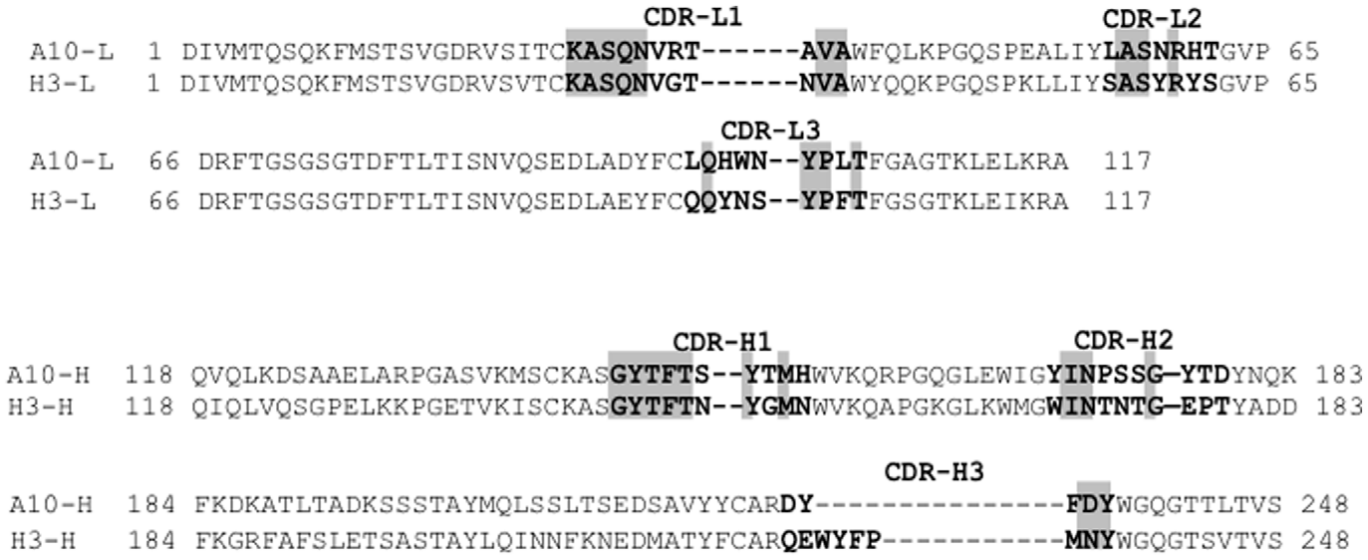
Amino acid sequences of anti-rNcSAG1 antibody variable regions. The amino acid numbers are according to the Kabat numbering scheme. The amino acids of CDR are bolded, and the identified amino acids in CDR are highlighted.

Heavy and light chain variable regions of A10 and H3 were sequenced and queried against entries in several databases, there is no antibody with the same sequence was found, suggesting A10 and H3 may be novel antibodies. Sequences of A10 and H3 were compared each other, and the identity of A10 and H3 (variable region) was 62% (Figure 7). However, their complementarity-determining regions (CDRs) showed a significant difference between A10 and H3.

The model structures of A10 and H3 were built using WAM antibody modeling server (http://antibody.bath.ac.uk) (Figure S4). Although the CDRL3 of two antibodies overlapped each other very well, the positions of CDRH3s are quite different, suggesting they might bind to different epitope of NcSAG1.

## Discussion

In this study, the variable region genes for heavy and light chains of two murine antibodies against rNcSAG1 were cloned successfully. The sequences of both antibodies were identified to be novel, and both antibodies showed high affinity against rNcSAG1. In immunofluorescence assays, both A10 and H3 Fabs bound to *N. caninum* parasites. Binding of A10 and H3 antibodies to rNcSAG1 was also inhibited by anti-*N. caninum* antibodies in the neosporosis-positive cattle sera, suggesting they might bind to the same epitopes of NcSAG1with those anti-*N. caninum* antibodies of bovine. However, in the inhibition experiments, the inhibitory capacity of the *Neospora*-positive serum sample was limited. This means that there were many kinds of anti-NcSAG1antibodies with different epitopes in the neosporosis-positive cattle serum. This is the reason why only about 60% binding capacity of the Fabs was inhibited, although the positive sera were diluted in 10 times.

Monoclonal antibodies are now established as a key therapeutic modality to a range of disease. Owing to the ability of these agents to selectively target tumor cells, cancer has been a major focus of development programs for monoclonal antibodies [20]. Phage display is a very useful technology to generate antibodies with affinities comparable to those obtained using traditional hybridoma technology. In general, two kinds of libraries, immune or naïve libraries can be constructed for selection, and the affinity of antibodies isolated from immune library are higher than those from naïve ones [21]. In this study, we employed the variable genes of antibodies from immunized mice, constructed an immune antibody library, and obtained two antibodies. However, the affinity of A10 and H3 may be limited because it depends on the size of library, because to an extent, the affinities of antibodies obtained from library are decided on the types and the sizes of libraries. By using A10 and H3 as a basis for construction and selection of libraries where variable gene is diversified, the affinity of A10 and H3 can be further increased to levels unobtainable in the immune system [22], [23]. When the amino acid sequences and modeling structures of A10 and H3 were compared, they have only low identity in the CDR regions, suggesting they may bind to different epitope of NcSAG1.

So far, many detection methods for neosporosis have been developed. Most of them focused on serological check, the detection of antibodies in serum sample of cattle. Another is a microscopic observation to check whether parasite is present in the tissue of cattle or not. Our study provides a novel method to detect parasite directly without microscopic observation that may not be available in small farms or animal hospitals. The detection of *N. caninum* parasites was established by sandwich ELISA using the monoclonal antibodies A10 and H3. This is the first sandwich method for detection of *N. caninum* parasites and may be used for monitoring them in dairy farms. When a tissue extract of suspicious cattle are available, the parasite can be detected easily. However, those tests only could be done after infection occurred. In *Neospora* transfection cycle, there are three stages, tachyzoite, tissue cyst, and oocyst. The tachyzoite and tissue cyst of the parasite are intracellular, but oocyst exits in the feces of dog. Dogs pass the oocyst to food or water that is taken in by cattle, which is the cause of infection. This method developed in this study is helpful to detect and to monitor the oocyst in farm environment, which leads to keep cattle away from the oocyst. This might be more important in view of disease prevention. Furthermore, the NcSAG1 of *N. caninum*, which was used as an immunogen in this study, only has 53% similarity with SAG1 protein of *Toxoplasma gondii* that is most close to *N. caninum.* However, since there is no cross-reactivity between those two parasites [8], this method might has a high reliability on detection of *N. caninum* parasites.

Comparing the CDR sequences between A10 and H3 antibodies, they might have different epitopes on the NcSAG1 protein. This leads to the novel sandwich ELISA possible for detection of *N. caninum*, because at least two antibodies against different epitopes are necessary. Nowadays, many antibody drugs for human beings have been developed and obtained approval from governments, but there are few for animals. Those antibodies also provide a possibility for development of antibody drugs for infectious diseases of animals.

## Materials and Methods

### Materials

The *N. caninum* Nc-Liv and Vero cell (No. CCL-81) strains were purchased from ATCC (No. 50845, Rockville, MD, USA). The *E. coli* strains DH5α for general cloning, XL10-Gold for cloning and amplification of phagemid, and TG-1 for displaying antibody on M13 phage were purchased from Agilent Technologies (La Jolla, CA, USA). Phagemid pDong1/Fab [24], helper phage KM13 and non-suppressor *E. coli* strain HB2151 were provided by Dr. H. Ueda of The University of Tokyo. Two inbred BALB/c mice, 5–6 weeks old, weighing 20–25 g (Japan SLC, Inc. Hamamatsu, Shizuoka, Japan), were maintained in the Animal House, Shizuoka University and used for the present study. Restriction and modification enzymes were purchased from Takara-Bio (Shiga, Japan), Toyobo (Osaka, Japan), Roche Diagnostics (Tokyo, Japan), or New England Biolabs (Ipswich, MA, USA). Oligonucleotides were synthesized by either Operon (Tokyo, Japan) or Invitrogen (Tokyo, Japan). Other chemicals, reagents and antibodies, unless otherwise indicated, were obtained from Sigma-Aldrich (St Louis, MO, USA) or Wako Pure Chem. (Osaka, Japan).

### Immunization of Mice with rNcSAG1 and Detection of NcSAG1-specific Antibodies in Sera Samples of Mice

Recombinant NcSAG1 was expressed in silkworm with a *Bombyx mori* nucleopolyhedrovirus bacmid system and purified [25]. Mice were immunized four times at 2-week intervals with 51 µg of rNcSAG1 by the subcutaneous route. To increase the efficiency of immunization, protein solutions are emulsified with a Freund’s complete adjuvant (Rockland Immunochem. Inc., Gilbertsville, PA, USA). After the last immunization, blood samples are taken by tale bleeding. Sera were separated by centrifuge at 3000 g for 10 min and were used to check the rNcSAG1-specific antibodies. The experiments with mice were carried out in strict accordance with the recommendations in the Guide for the Care and Use of Laboratory Animals of Shizuoka University. The protocol was approved by the Committee on the Ethics of Animal Experiments of Shizuoka University (Permit Number: 24–11).

The rNcSAG1-specific antibodies in sera samples of mice were confirmed using an indirect ELISA. Each well of a microplate was coated with 100 ng of recombinant protein in 100 µl of phosphate-buffered saline (PBS: KH_2_PO_4_, 1.47 mM; Na_2_HPO_4_, 8.10 mM; NaCl, 136.89 mM; KCl, 2.68 mM) overnight at 4°C. After removal of the solution, 200 µl of MPBS (2% skimmed milk in PBS) was added to each well and incubated for 2 h at 25°C. After incubation, each well was washed three times with PBST (0.1% Tween 20 in PBS); 100 µl of sera samples from *N. caninum* protein-infected mice, diluted 10^3^-, 10^4^-, 10^5^-, and 10^6^-fold in MPBS, were added, and the plates were incubated for 1 h at 25°C. Each well was washed three times with PBST and incubated with 100 µl/well of 5000-fold diluted ECL™ Anti-mouse IgG, Horseradish Peroxidase linked whole antibody (from sheep; GE Healthcare UK limited) in MPBS. The bound anti-NcSAG1 antibodies were detected by the addition of 3,3′,5,5′-tetramethylbenzidine substrate solution (TMBZ; 100 µg/ml TMBZ and 0.04 µl/ml H_2_O_2_ in 100 mM NaOAc, pH 6.0; Sigma-Aldrich) after washing to remove the unbound secondary antibodies. BSA was also immobilized as a control. The immunized mice were sacrificed to obtain the spleen after the presence of NcSAG1-specific antibodies was confirmed.

### Cloning of Antibody Variable Region Genes and Construction of Fab Fragment Libraries

The DNA fragments for V_H_ and V_L_ were prepared from total RNA derived from the spleen using PrimeScript One Step RT Kit ver. 2 (Takara, Tokyo, Japan) according to the manufacturer’s protocol. The mouse V_H_/V_L_-specific primers are synthesized based on the common antibody primer sequences (Table S1). The PCR products were then purified using Illustra™ GFX™ PCR DNA and Gel Band Purification kit (GE Healthcare). The purified V_L_ fragments were digested with restriction enzymes *Sal*I and *Not*I, again purified and ligated using T4 DNA ligase at 16°C for 1 h with a phagemid pDong1/Fab, which had been digested with the same enzymes. After confirmation of the inserted V_L_ sequence of several clones out of the obtained ones, the V_H_ fragments were inserted into the V_L_-inserted phagemid library using restriction enzymes *Sfi*I and *Xho*I. Electroporation-competent *E. coli* TG-1 cells were transformed with the ligation product and plated on 2YTAG agar (16 g/l tryptone, 10 g/l yeast extract, 5 g/l NaCl, pH 7.2, supplemented with 100 µg/ml ampicillin, 1% glucose, and 1.5% agar) plates overnight at 37°C. The size of library was estimated from the number of colonies on the plate.

### Fab Antibody Phage Display

*E. coli* TG-1 cells, transformed with the phagemid, were cultivated in 4 ml of 2YTAG overnight at 37°C. Ten milliliters of 2YTAG was inoculated with 100 µl of the overnight culture at 37°C with shaking at 200 rpm until OD_600_ reached around 0.5, when helper phage KM13 [16] was added with a multiplicity of infection (MOI) of 20. After incubation at 37°C for 30 min without shaking, the culture was centrifuged at 3700 g for 15 min. Then, the *E. coli* pellet was resuspended in 50 ml of 2YTAK (2YT medium containing 100 µg/ml ampicillin and 50 µg/ml kanamycin) and incubated overnight with shaking at 30°C. The overnight culture was centrifuged at 10800 g for 30 min. Ten milliliters of PEG/NaCl (20% polyethylene glycol 6000, 2.5 M NaCl) was added to 40 ml of supernatant, and the mixture was incubated on ice for 1 h. After incubation, the mixture was centrifuged at 6000 g for 30 min. The pellet was resuspended in 2 ml of PBS and centrifuged at 15000 g for 10 min to pellet cell debris, and the supernatant was collected as Fab-displaying phage solution.

### Phage ELISA

The antigen-binding capacity of phage-displayed Fab fragments was tested with phage ELISA. The microplates (NUNC, Langenselbold, Germany) were coated overnight with 100 µl of rNcSAG1 (0.5 µg/ml) per well or 10 µg/ml of BSA (Sigma-Aldrich) in PBS at 4°C. Plate was blocked at 25°C for 2 h with 2% MPBS, washed three times with PBST, and incubated with 100 µl/well of MPBS containing 10^9^–10^10^ colony form unit (cfu) of Fab-displaying phage at 25°C for 1 h. The plate was washed three times with PBST and incubated with 100 µl/well of 5000-fold diluted HRP/anti-M13 monoclonal conjugate (GE Healthcare) in MPBS at 25°C for 1 h. The plate was then washed three times with PBST and developed with 100 µl/well TMBZ solution. After incubation for appropriate time, the reaction was stopped by adding 50 µl/well of 10% sulfuric acid, and the absorbance was read using a Model 680 microplate reader (Bio-Rad, Tokyo, Japan) at 450 nm with 655 nm as a control.

### Selection of rNcSAG1-specific Antibody Phage from Phage Library

Antibody selection from the phage display library was performed on microplates on which 100 µl of rNcSAG1 (1 µg/ml in PBS) was immobilized at 4°C overnight. The microplates were washed with 200 µl of PBST three times, then blocked with MPBS for 2 h, followed by adding 100 µl of phage solution (10^12^ cfu/ml in PBS) and incubated for 1 h at 25°C. After washing again with PBST for six times, phages bound to the microplates were eluted with 100 µl of 1.0 mg/ml TPCK-treated trypsin (Sigma-Aldrich) in PBS. *E. coli* TG-1 cells (OD_600_ = 0.5 in 700 µl culture) were infected with 100 µl of eluted phage solution and cultured in 10 ml of 2YTAG medium at 37°C with shaking at 200 rpm. When OD_600_ reached 0.5, the KM13 helper phage was added at a MOI of 20, and incubated for 30 min at 37°C without shaking. After being centrifuged at 3700 g for 10 min, the pellet was resuspended in 50 ml of 2YTAK medium and incubated with vigorous shaking at 30°C overnight. The culture supernatant was prepared by centrifugation at 10800 g for 30 min, and phages were precipitated with 0.2 volume of PEG/NaCl on ice for 1 h. After centrifugation at 6000 g for 30 min, the pellet was resuspended in PBS and used as a source of round 1 (R1) phage. Round 2 (R2) antibody-selection from R1 phage was performed as described above and R2 phage was obtained. From R2 phage library, round 3 (R3) selection was also carried out to make R3 phage. The enrichment of rNcSAG1 specific phage-antibody among R0 phage (original phage library), R1, R2, and R3 phages was confirmed with a polyclonal phage ELISA.

After the increase of binding capability of phage was confirmed, ninety-six *E. coli*-infected clones at the 3^rd^ biopanning were picked up, cultivated for making monoclonal phage. A phage ELISA was performed for 96 individual clone to select rNcSAG1-specific phage-antibodies. Nucleotide sequence of positive clones was analyzed on a Beckman sequencer (CEQ8000, Beckman Coulter, Inc., Brea CA, USA) and Genetyx software.

### Competitive ELISA

For evaluation of binding capacity of positive clones to rNcSAG1 and the inhibition of anti-*N. caninum* antibodies in cattle serum to the binding, competitive ELISAs were performed. Two groups of cattle sera samples containing one negative sample and one positive sample were employed. One was the component of a commercial *N. caninum* iscom ELISA kit purchased from SVANOVA Biotech AB (SVANOVA Biotech AB, Boehringer Ingelheim Svanova, Uppsala, Sweden), and the other group was from Tobu Livestock Diagnostic Center of Shizuoka Prefecture of Japan. Fab-phage, mixed with series diluted rNcSAG1 or serial diluted sera from infected or healthy cattle, was added to microplate wells on which rNcSAG1 was coated after being blocked with MPBS. For the cattle sera from Tobu Livestock Diagnostic Center of Shizuoka Prefecture of Japan, 10-folded diluted samples were used for test. After incubation and washing, 5000-fold diluted HRP/anti-M13 monoclonal conjugate was added. Detection was performed according to protocol described in the Phage ELISA section.

### Expression of Fabs of Monoclonal Antibody

Fabs of positive antibodies were expressed with a nonsuppressor strain HB2151. For convenient expression of Fab, the TAG stop codon was designed between V_H_-C_H_1 gene and gene III of M13 in pDong1/Fab [24]. With this design, Fab can be expressed as a fusion protein with surface protein gPIII of M13 phage, resulting in the display of Fab on phage. However, when nonsuppressor strain HB2151 was used, Fab will be expressed as a soluble fragment independently. In brief, 200 µl of exponentially growing HB2151 was infected with 10^9^ cfu of phage for 30 min at 37°C. Infected-*E. coli* cells were pelleted by centrifuge at 5000 g for 10 min and resuspended in 4 ml 2YT medium containing 100 µg/ml of ampicillin (2YTA) and cultivated for 3 h at 37°C. Four hundred milliliters of 2YTA medium was inoculated with the 4-ml culture and cultivated at 37°C with shaking. Once the OD_660_ reached 0.5, and then isopropyl β-D-thiogalactoside (IPTG) with a final concentration of 1 mM was added and cultivated further overnight at 30°C.

The *E. coli* cells were harvested by centrifugation at 4000 × g for 20 min at 4°C. The periplasmic fraction was extracted according to a general protocol. Supernatant of the culture was also collected and precipitated by adding ammonium sulfate at a final concentration of 75% (w/w). His-tagged Fabs were purified from the periplasmic fraction and concentrated supernatant with TALON Co^2+^-immobilized resin (Takara-Bio) according to the instructions provided by the manufacturer. Because one-step purification was not enough, Fabs were furthermore purified with an anti-FLAG M2 affinity gel (Sigma-Aldrich) according to the instructions provided by the manufacturer. The purified Fabs of A10 and H3 were analyzed using polyacrylamide gel electrophoresis as described by Laemmli [26].

### Immunofluorescence Assay of *N. caninum* with Monoclonal Antibodies

Vero cells infected with *N. caninum* tachyzoites were maintained in a CO_2_ incubator at 35°C. Freshly purified *N. caninum* tachyzoites from Vero cells were fixed on a slide glass, permeabilized, and blocked with 8% BSA in PBS. Immunolabelling was carried out by using purified A10 (10 µg/ml), H3 (10 µg/ml), and commercial anti-*Neospora* antibodies (1 µg/ml). A sample without any primary antibody was used as a negative control. After one-hour incubation, anti-FLAG tag antibody (1 µg/ml) was added to samples on the slide glass. Alexa 594 conjugated anti-Mouse IgG was then added to all the samples after incubation and washed the samples with PBS. All samples were viewed on a confocal microscope (LSM 700, Carl-Zeiss, Oberkochen, Germany), and images were processed by employing the software ZEN lite 2009 (ZEISS).

### Detection of *N. caninum* Parasites

A sandwich ELISA was designed to detect *N. caninum* parasites directly. One hundred microliter of PBS solution containing 10 µg/ml of A10 Fab was added to wells of a microplate and incubated at 4°C for overnight. After blocking with 200 µl of MPBS for 2 h, 100 µl of serial diluted solutions containing 1.563×10^3^, 3.125×10^3^, 6.25×10^3^, 1.25×10^4^, 2.5×10^4^ parasites, respectively, and a solution without parasite, were then added to each well and incubated at 25°C for 2 h. The parasite number was counted by microscopic observation. After washing three times with PBST, 100 µl/well of Fab H3 displaying phage was then added at a concentration of 10^9^ cfu/well and incubated at room temperature for 2 h. The wells were washed three times with PBST and incubated with 100 µl/well of 5000-fold diluted HRP/anti-M13 monoclonal antibody conjugate (GE Healthcare) in MPBS at 25°C for 1 h. TMBZ solution was added to each well to detect H3-parasites-A10 complex. Assays with 0, 0.625, 1.25, 2.5, 5 and 10 µg/ml of BSA instead of parasite were also carried out as negative controls. The LOD for *N. caninum* was obtained as the estimated parasite number that shows the mean blank value plus 3 SD.

## Supporting Information

Figure S1 Scheme for the development of murine anti-NcSAG1 antibodies. The variable region genes of antibodies were amplified from cDNA, which was transcribed from total RNA extracted from spleen cells of immunized mice and cloned into a phagemid pDong1/Fab. By transformation of *E. coli* with pDong1-containing antibody gene, a phage displayed Fab library was made and used for monoclonal antibody selection. V_H_: variable region gene of heavy chain of antibody; V_L_: variable region gene of light chain of antibody; C_H_1: constant region gene of heavy chain of antibody; C_L_: constant region gene of light chain of antibody.
(TIFF)

Figure S2 Confirmation of anti-rNcSAG1 antibodies in immunized mouse. After immunization, anti-NcSAG1 antibodies in the serum of mouse were confirmed using an ELISA with immobilization of BSA or rNcSAG1 on a microplate. After blocking with PBS containing skimmed milk, microplate was added with diluted mouse sera samples in 10^3^, 10^4^, 10^5^, and 10^6^ times. Then, the ECLTM Anti-mouse IgG, Horseradish Peroxidase linked whole antibody (from sheep) was added. After incubation at room temperature for 1 h, the assay was carried out.
(TIFF)

Figure S3 Inhibition of A10 and H3 antibody binding to rNcSAG1 with anti-*N. caninum* antibodies in neosporosis-positive serum. Sera samples from healthy cattle (negative) and *N. caninum*-infected cattle (positive), which were different from those used in Figure 6, were provided by Tobu Livestock Disease Diagnostic Center of Japan. These sera were used to inhibit the binding of A10 or H3 to immobilized rNcSAG1on a microplate (n = 3).
(TIFF)

Figure S4 Molecular model of the variable regions of antibodies A10 and H3. Structures were built using WAM antibody modeling server. A10 is shown in green, and the blue parts stand for the A10-CDRH3 and A10-CDRL3; H3 is in red, and dark red parts stand for the H3-CDRH3 and H3-CDRL3.
(TIFF)

Table S1 Primers used in this study.
(DOCX)
