# Supplementary figures and images for: Development of Two Murine Antibodies against Neospora caninum Using Phage Display Technology and Application on the Detection of N. caninum
Source: PLoS One. 2013 Jan 8;8(1):e53264. doi: 10.1371/journal.pone.0053264 (PMC3540087; doi:10.1371/journal.pone.0053264)

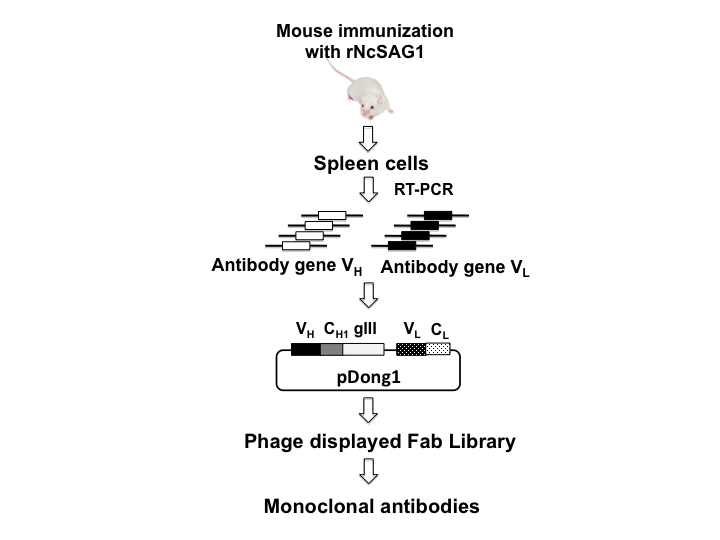

Supplement: Figure S1 — Scheme for the development of murine anti-NcSAG1 antibodies. The variable region genes of antibodies were amplified from cDNA, which was transcribed from total RNA extracted from spleen cells of immunized mice and cloned into a phagemid pDong1/Fab. By transformation of E. coli with pDong1-containing antibody gene, a phage displayed Fab library was made and used for monoclonal antibody selection. VH: variable region gene of heavy chain of antibody; VL: variable region gene of light chain of antibody; CH1: constant region gene of heavy chain of antibody; CL: constant region gene of light chain of antibody. (TIFF) [file pone.0053264.s001.tiff]

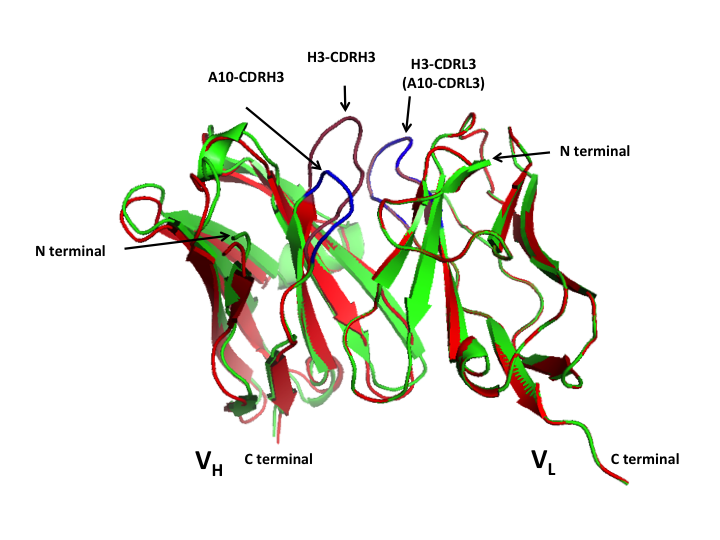

Supplement: Figure S4 — Molecular model of the variable regions of antibodies A10 and H3. Structures were built using WAM antibody modeling server. A10 is shown in green, and the blue parts stand for the A10-CDRH3 and A10-CDRL3; H3 is in red, and dark red parts stand for the H3-CDRH3 and H3-CDRL3. (TIFF) [file pone.0053264.s004.tiff]
