# Supplementary material for: Development of Two Murine Antibodies against Neospora caninum Using Phage Display Technology and Application on the Detection of N. caninum
Source: PLoS One. 2013 Jan 8;8(1):e53264. doi: 10.1371/journal.pone.0053264 (PMC3540087; doi:10.1371/journal.pone.0053264)
Supplement: Table S1 — Primers used in this study. (DOCX) [file pone.0053264.s005.docx]

**Table S1.** Primers used in this study

| **Primer name** | **Sequence (5’-3’)^*^** |
| --- | --- |
| VH1 | CTTTCTATGCGGCCCAGCCGGCCATGGCCSAGGTYCAGCTBCAGCAGTC |
| VH2 | CTTTCTATGCGGCCCAGCCGGCCATGGCCCAGGTTCACCTGCAGCARTC |
| VH3 | CTTTCTATGCGGCCCAGCCGGCCATGGCCCAGGTRCAGCTGAAGGAGTC |
| VH4 | CTTTCTATGCGGCCCAGCCGGCCATGGCCCAGGTCCAACTVCAGCARCC |
| VH5 | CTTTCTATGCGGCCCAGCCGGCCATGGCCCAGATCCAGTTGGTVCAGTC |
| VH6 | CTTTCTATGCGGCCCAGCCGGCCATGGCCCAGGTGCAGCTGAAGSASTC |
| VH7 | CTTTCTATGCGGCCCAGCCGGCCATGGCCGAGGTGCAGSKGGTGGAGTC |
| VH8 | CTTTCTATGCGGCCCAGCCGGCCATGGCCGAGGTGAARSTTGAGGAGTC |
| VH9 | CTTTCTATGCGGCCCAGCCGGCCATGGCCGAKGTSVAGCTTCAGGAGTC |
| VH10 | CTTTCTATGCGGCCCAGCCGGCCATGGCCGAGGTGAASSTGGTGGAATC |
| VH11 | CTTTCTATGCGGCCCAGCCGGCCATGGCCGAGGTGAAGCTGRTGGARTC |
| VH12 | CTTTCTATGCGGCCCAGCCGGCCATGGCCGARGTGAAGCTGRTGGAGTC |
| VH13 | CTTTCTATGCGGCCCAGCCGGCCATGGCCGAAGTGCAGCTGTTGGAGAC |
| VH14 | CTTTCTATGCGGCCCAGCCGGCCATGGCCGARGTGAAGCTTCTCSAGTC |
| VH15 | CTTTCTATGCGGCCCAGCCGGCCATGGCCCARGTTACTCTGAAAGAGT |
| JH1 | ACTGCTCGAGACGGTGACCGTGGTCCC |
| JH2 | ACTGCTCGAGACTGTGAGAGTGGTGCC |
| JH3 | ACTGCTCGAGACAGTGACSCAGAGTCCC |
| JH4 | ACTGCTCGAGACGGTGACTGAGGTTCC |
| VK1 | TATTCGTCGACGGATATTGTGATGACBCAGDC |
| VK2 | TATTCGTCGACGGATRTTKTGATGACCCARAC |
| VK3 | TATTCGTCGACGGAAAATGTGCTCACCCAGTC |
| VK4 | TATTCGTCGACGGAYATTGTGATGACACAGTC |
| VK5 | TATTCGTCGACGGACATCCAGATGACACAGAC |
| VK6 | TATTCGTCGACGGAYATTGTGCTSACYCARTC |
| VK7 | TATTCGTCGACGGACATCCAGATGACYCARTC |
| VK8 | TATTCGTCGACGCAAATTGTTCTCACCCAGTC |
| JK1/2 | TTCTCGTGCGGCCGCACGTTTKATTTCCAGCTTGG |
| JK4 | TTCTCGTGCGGCCGCACGTTTTATTTCCAACTTTG |
| JK5 | TTCTCGTGCGGCCGCACGTTTCAGCTCCAGCTTGG |

^*^The underlined sequences indicate the article registration enzyme sites.
